# Turkish Validity and Reliability of the Self-Applied Acute Stress Scale (EASE) for Healthcare Providers

**DOI:** 10.1155/2024/7673595

**Published:** 2024-06-10

**Authors:** Çağlar Şimşek, Melike Mercan Baspinar

**Affiliations:** ^1^ Department of Nursing University of Health Sciences Taksim Training and Research Hospital, Taksim, Sıraselviler Street No. 48, Beyoglu 34433, Istanbul, Türkiye; ^2^ Department of Family Medicine University of Health Sciences Gaziosmanpasa Training and Research Hospital, Osmanbey Street, Gaziosmanpasa 34255, Istanbul, Türkiye

## Abstract

**Background:**

Acute stress induced by a sudden burden of emergency conditions and traumatic events, such as wars, earthquakes, situations requiring isolation, pandemics, and disasters, can have pathological consequences on healthcare providers (HCPs) if not diagnosed early. Therefore, the objective of this investigation is to culturally validate the self-administered Acute Stress Scale (EASE) in the Turkish context.

**Method:**

The study consisted of 127 HCPs working with COVID-19 patients in services and clinics during the pandemic. The individual information form and EASE were used for data collection. Confirmatory factor analysis (CFA) was used to test the factor structure of the EASE.

**Results:**

All the statistical procedures showed that the Turkish version of the EASE scale is a valid and reliable measurement tool for the Turkish culture. The content validity index (CVI = 0.84), intraclass correlation coefficient (ICC = 0.912), and model fit indices (*χ*^2^/df = 1.826, RMSEA = 0.083, CFI = 0.947, NFI = 0.893, GFI = 0.905) explained two-factor structure.

**Conclusion:**

Institutional approaches are necessary to support the psychological needs of HCPs. The Turkish version of the EASE scale demonstrated adequate reliability and validity properties. The scale could provide appropriate support during the early stages of acute stress among HCPs related to needs during isolation conditions or unexpected emergencies such as recent pandemics and epidemics in the future.

## 1. Introduction

Experiencing a traumatic event is remarkably common, with estimated lifetime self-reported exposure rates reaching 70.4% globally [1]. Acute stress disorder (ASD) is an unsettling response that typically emerges shortly after a traumatic event, persists for nearly a month, and can lead to a decline in overall well-being [2]. The aim of treatment for ASD is to reduce the severity of symptoms and prevent the development of post-traumatic stress disorder (PTSD). The observed positive correlation between healthcare providers (HCPs) and their preparedness levels suggests a potential pathway for fostering improvements in community health following an emergency [3]. Healthcare providers affected by the psychiatric health status of infected and uninfected patients were also victims of the COVID-19 pandemic [4]. Anxiety and depression scores were significantly higher among healthcare teams during the pandemic, with a higher prevalence of mental disorders among HCPs closer to infected patients [5]. HCPs at a high risk during the pandemic experienced higher levels of stress that may have persisted beyond the pandemic period. High-risk healthcare workers can have greater depression ratings and increased perceived stress even after the crisis subsides. HCPs at hospitals treating infected patients during a pandemic may experience higher levels of stress and PTSD than those at neighboring hospitals treating noninfected patients [6].

Studies show that the prevalence of PTSD ranged from 30% to 40% among immediate victims, 10% to 20% among healthcare workers, and 5% to 10% among the general population [7]. Illness affects the psyche of HCPs working on the front lines during a pandemic, increasing their risk of developing psychiatric symptoms [8].

Turkey confirmed its first COVID-19 case on March 11, 2020 [8]. Findings by Turkish physicians suggest that COVID-19 increased psychological stress in HCPs during the pandemic as well as associated physical symptoms such as personal stress, anxiety, panic attacks, depressive tendencies, and sleep disturbances [9]. Increased emotional and anxiety responses and symptoms, including post-traumatic stress, can be expected among HCPs who see parents and friends threatened [10].

The self-applied Acute Stress Scale (EASE) is a new tool to early detect distress among HCPs in emergency situations such as pandemic, infectious diseases, wars, and earthquakes [4]. EASE has English version, Spanish-Spain version, Spanish-Latin American version, and Brazilian Portuguese version [4]. Our study is aimed at culturally adapting the EASE to the Turkish language.

## 2. Methods

### 2.1. Permissions, Translation, and Adaptation of EASE Items

This psychometric study used a cross-sectional design. To adapt the EASE scale to the Turkish culture, we obtained the permission and information on the original version from the original researcher Jose Joaquin Mira. The Clinical Research Ethics Committee of Gaziosmanpaşa Training and Research Hospital accepted the study protocol on April 28, 2021 (approval no. 271).

The English version of the EASE scale was translated and adopted into the Turkish language in line with the recommendations in the literature [11]. Step 1 was forward translation. A professional translator who was a native English speaker and fluent in Turkish translated the original EASE into Turkish. Step 2 was back translation. An instructor working in Uskudar University's Foreign Languages Department who was also fluent in English translated the English EASE back into Turkish. Step 3 was pretesting and cognitive debriefing.

### 2.2. Face Validity

The scale was sent to a total of 15 specialists for assessment of understanding and representativeness of scale items by health colleagues. This expert committee had three mental health and psychiatric nursing faculty members, two psychology department faculty members, five psychologists, two anesthesiologists, two family physicians, and one pulmonary disease specialist. Experts were asked to evaluate items for the calculation of the content validity index (significance > 0.70) [12]. The authors discussed and considered potential changes to the translated introduction and items.

### 2.3. Instrument

EASE is a 10-point scale with strong reliability (Cronbach's alpha = 0.85) and validity based on a study of 228 HCPs in the Spanish public health system. Total scores range from 0 to 30, where 0–9 indicates good emotional regulation, 10–14 indicates emotional distress, 15–24 indicates emotional overload, and 25 and above indicates extreme acute stress. The two-factor structure of the scale includes affective responses as well as fear and anxiety responses [4, 13]. Confirmatory factor analysis (CFA) confirmed the underlying two-factor structure and model fit indices, *χ*^2^/df = 9.04, RMSEA = 0.085, CFI = 0.92, and GFI = 0.93, explaining 55% of the variance [4]. Ten items in the scale are as follows: item 1: I cannot help but think of recent critical situations. I cannot get out of work; item 2: I have completely lost the taste for things that gave me peace of mind; item 3: I keep my distance, I resent dealing with people, and I am irascible even at home; item 4: I feel that I am neglecting many people who need my help; item 5: I have difficulty thinking and making decisions, I have many doubts, and I have entered a kind of emotional blockage; item 6: I feel intense physiological reactions (shock, sweating, dizziness, shortness of breath, insomnia, etc.) related to the current crisis; item 7: I feel on permanent alert. I believe that my reactions now put other patients, my colleagues, or myself at risk; item 8: Worrying about not getting sick causes me a strain that is hard to bear; Item 9: I am afraid I am going to infect my family; and item 10: I have difficulty empathizing with patients' suffering or connecting with their situation (emotional distancing and emotional anesthesia) [4]. Turkish and English versions of EASE are presented in Tables 1 and 2.

### 2.4. Participants and Selection

An online sample size calculator for structural equation model was used to calculate minimum sample size [14, 15]. Assuming the original structure of the questionnaire (2 subdimensions, 10 items) and a moderate effect (0.3), a test was conducted with a power of 0.8 and a significance level of *α* = 0.05. The minimum sample size for the model was 100 individuals [14]. The research study sample reached to final 127 HCPs working in a training and research hospital located on the European side of Istanbul between June 1, 2021, and July 1, 2021. The inclusion criteria were working with infected patients in COVID-19 services and clinics of a tertiary hospital during the outbreak and fluency in the Turkish language. HCPs were informed about the procedure, and their written consent was obtained.

### 2.5. Statistical Analysis

All statistical analyses were performed using SPSS 25.0 (IBM SPSS Statistics 25 software (Armonk, NY: IBM Corp.)) and AMOS 23.0 (IBM SPSS AMOS 23.0 software (Armonk, NY: IBM Corp.)). Continuous variables were expressed as mean ± standard deviation (SD) and median (25th and 75th percentiles), and categorical variables were expressed as frequencies and percentages. We used the histograms and box plots to obtain information about the distribution of scale and item scores. To ensure the data's suitability for a factor analysis, we conducted Kaiser-Meyer-Olkin (KMO) and Bartlett's tests to compare the observed correlation matrix to the identity matrix. Test-retest reliability was assessed using the intraclass correlation coefficient (ICC) and the Wilcoxon signed rank test over a two-week interval. We evaluated the internal consistency of instrument reliability using Cronbach's alpha, with a desirable level of >0.70. We also performed a confirmatory factor analysis (CFA) to verify that the Turkish version's factors loaded into the correct constructs [16]. We used several goodness-of-fit indicators, including root mean square error of approximation (RMSEA), where 0.05 to 0.08 indicates an acceptable fit; comparative fit index (CFI), where ≥0.90 indicates an acceptable fit; and chi-square absolute and predictive fit, where a nonsignificant *χ*2 indicates a good fit [17, 18]. Statistical significance was set at *p* < 0.05.

## 3. Results

As shown in Table 3, the sample's median age and job experience were 37 years old and 13 years, respectively. The COVID-19 vaccination rate was 88.19% (*n* = 112), and COVID-19 disease history was positive in 25.20% (*n* = 32) of the sample. The first test total score was 10.31 ± 5.68, and the total retest score was 9.86 ± 5.04. A content validity index (CVI) value of 0.84 was obtained. Since the CVI value was greater than the critical CVI value of 0.6 for 15 experts, the CVI value obtained was deemed acceptable [12].

The KMO value was higher than 0.60 (KMO = 0.899), and the Bartlett's test was significant (*χ*^2^ = 574.006, df = 45, *p* < 0.001), which means that the sample size and correlation matrix were adequate for a factor analysis [18].

The Cronbach's alpha value of the first test was 0.886, while the retest Cronbach's alpha value was 0.862. The compatibility of the answers given in the test and retest applications was compared with the ICC and showed that reliable responses were given to all items. ICC coefficients were in a good (0.75–0.90) and very good (>0.90) range based on Koo and Li [19]. ICC value of scale was 0.912 for our study. Cronbach's alpha to estimate the internal consistency of the instrument's reliability was within an acceptable level of >0.70 [12, 18]. The results of the ICC evaluation are summarized in Table 4.

The Cronbach's alpha values obtained in both the test and retest applications were found to be high (>0.70). Item-total correlations ranged from 0.390 to 0.740 for 10 items. An item-total correlation coefficient of 0.30 and above is interpreted as good for reliability. In addition, when the items were removed from the scale (Table 5), it was evident that there was no need for any item to be removed from the scale.

Tables 6 and 7 summarize the results of the confirmatory factor analysis and factor loads based on the original version of the scale's factor structure. In Table 6, the model fit indices (*χ*^2^/df = 1.826, RMSEA = 0.083, CFI = 0.947, NFI = 0.893, GFI = 0.905) showed two-factor structure. They were all acceptable [18]. In Table 7, items 1, 2, 3, 4, 5, and 10 were obtained in the first subdimension while items 6, 7, 8, and 9 were found in the second subdimension. The results show that the values of the model fit indices of the confirmatory factor analysis were all good results. When the factor loads of the items were examined, we found that all the results were usable. All values obtained in the study were found to be at or close to acceptable levels.

**Figure 1:**
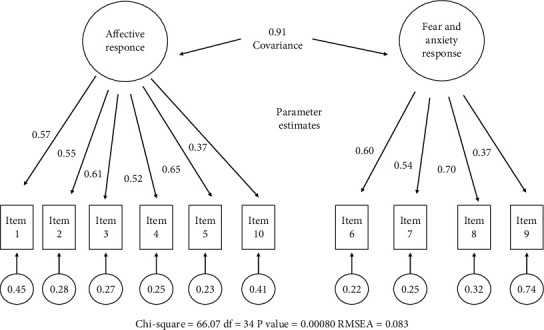
Path diagram of the confirmatory factor analysis. Standardized weights and measurement errors of each item of the EASE.

Figure 1 illustrates the standardized weights and measurement errors of each item of the EASE with a path diagram of the confirmatory factor analysis.

Table 8 summarizes the Turkish results of the original EASE scale measurement ranges and subdimensions. Among the participants, 47.24% (*n* = 60) showed a good emotional adjustment, 19.69% (*n* = 25) an emotional overload, and 31.50% (*n* = 40) emotional distress. Total score was 10.31 ± 5.68 points.

## 4. Discussion

The psychological impact of pandemics on HCPs has been a topic of increasing concern. Pandemics can lead to traumatic stress disorders and acute stress reactions among HCPs [20]. This study was carried out between June 1, 2021, and July 1, 2021, and included individuals who have been working with COVID-19 patients in a pandemic hospital. The study is aimed at validating the EASE in Turkish and determining the distress related to working with COVID-19 patients among Turkish HCPs during the COVID-19 pandemic. According to our results, CVI (0.84), ICC (0.912) values, and model fit indices (*χ*^2^/df = 1.826, RMSEA = 0.083, CFI = 0.947, NFI = 0.893, GFI = 0.905) indicated that the Turkish EASE scale can be used to assess acute stress among Turkish HCPs in unexpected conditions such as a global pandemic.

Based on the European Centre for Disease Prevention and Control report on Spain, HCPs accounted for 20% of registered COVID-19 cases compared with 3.8% in China, 10% in Italy, and 3% in the USA during the first COVID-19 outbreak in 2020 [21]. As of May 9, 2021, Turkey had the fifth most COVID-19 cases in the world and had entered a nationwide lockdown; the total death was 43,029 with 5,031,332 reported infections [22]. Due to the increase in the need for isolation services for COVID-19 patients, institutions had to convert their clinics into isolation centers, and most of the hospitals were put into strict pandemic service [21, 23]. Stressors for HCPs included risk of being infected [24]. This change in time and pandemic episode caused distress among HCPs. As shown in a study from Turkey, by May 2020, 50% had mild and 17% had severe anxiety of study sample. The relationship between fear of death and disease transmission and anxiety levels was found to be significant [25]. Another study demonstrated that 29.6% of HCPs were psychologically influenced with mild to severe symptoms due to the outbreak. The psychological impact on HCPs may have occurred before the pandemic reached the hospitals, highlighting the need to utilize psychological measures as early as possible [25].

We aimed to validate a scale for the early protection of HCPs from acute distress based on the COVID-19 pandemic period. However, our study has several limitations. First, there was no COVID-19 vaccination during the original scale development study in Spain, whereas 88% of the Turkish sample's participants had vaccine protection against the disease, which might be a confounding factor for an acute stress/distress measurement. By February 2021, Turkey's vaccination percentages in the first 23 days were an encouraging 3.04%, higher than that of Italy (2.06%), Spain (2.90%), the UK (2.36%), and the USA (2.69%) [22].

Second, 25.2% of the participants of our study sample had the COVID-19, which might have affected their critical affective responses, fear, and anxiety levels. Xiao et al. conducted a study on acute stress reaction among medical staff during the COVID-19 outbreak in China to evaluate stress-related symptomatology—including negative mood, intrusion, dissociation, avoidance, and arousal—that was either initiated or worsened shortly after the event [26]. Uygur et al. found that COVID-19 worry was correlated with COVID-19 fear, COVID-19 anxiety, women, the presence of positive first-degree relatives, and the presence of chronic diseases [27]. The presence of chronic disease was also found to be significant in terms of COVID-19 anxiety in studies of Egren et al. and Süntar et al. [28, 29]. Hence, the absence of inquiry into the frequency of chronic diseases in our study represents a limitation in terms of the homogenization of our study group. However, the global scores of the study group in a Spain sampling were 10.0 ± 6.10 [13], which closely resembles our study's findings, wherein the mean scores were 10.31 ± 5.68 points. This indicates a similar level of emotional distress experienced by healthcare professionals (HCPs) in both contexts. Notably, the mean value of EASE suggests that Turkish HCPs underwent emotional distress, despite our study commencing one year after the outbreak of the pandemic.

### 4.1. Limitation

Several guidelines are recommended for establishing sufficient evidence of reliability and validity. For clinical trials, a minimum reliability threshold of 0.70 is recommended and sample sizes for testing should include at least 200 cases and results should be replicated in at least one additional sample [30]. Even if the reliability coefficient is acceptable, the study sample size in our study of 127 participants was a limitation of this study.

## 5. Conclusion

Outbreak and emergence of isolation conditions requiring protection are known to have a psychological impact on HCPs. Therefore, psychosocial interventions are crucial, particularly for high-risk frontline HCPs. The implementation of the EASE scale could play a significant role in the early detection of HCPs' needs by measuring their distress during future unexpected conditions, disease outbreaks, and isolation units.

Concern about disease transmission exists when working with all infectious diseases. For this reason, the EASE scale can be used not only for outbreaks but also for acute stress measurement in healthcare workers who have just started working with diseases such as tuberculosis and HIV, which require isolation conditions.

Consequently, future studies should focus on analyzing distress among HCPs in Turkey to identify practical measures aimed at reducing psychological stress and preventing future harm resulting from unexpected conditions.

## Figures and Tables

**Table 1:** English version of the EASE.

Self-administered Acute Stress Scale (EASE) for healthcare professionals				
Please answer the following questions according to the thoughts, emotions, sensations, and actions you are experiencing during these days of crisis	It is not happening to me (0)	It happens to me in concrete situations (1)	It often happens to me (2)	I am like this all the time (3)
*Affective responses*				
(1) I cannot help but think of recent critical situations. I cannot get out of work.				
(2) I have completely lost the taste for things that gave me peace of mind.				
(3) I keep my distance, I resent dealing with people, I am irascible even at home.				
(4) I feel that I am neglecting many people who need my help.				
(5) I have difficulty thinking and making decisions, I have many doubts, I have entered a kind of emotional blockage.				
(10) I have difficulty empathizing with patients' suffering or connecting with their situation (emotional distancing, emotional anesthesia).				

*Fear and anxiety responses*				
(6) I feel intense physiological reactions (shocks, sweating, dizziness, shortness of breath, insomnia, etc.) related to the current crisis situation.				
(7) I feel on permanent alert. I believe that my reactions now put other patients, my colleagues or myself at risk.				
(8) Worrying about not getting sick causes me a strain that is hard to bear.				
(9) I am afraid I am going to infect my family.				

**Table 2:** Turkish version of the EASE.

Sağlık Profesyonelleri için Kendi Kendine Uygulanabilen Akut Stres Ölçeği (EASE)				
Aşağıdaki soruları bu kriz günlerinde yaşadığınız düşünce, duygu, his ve eylemlere göre cevaplayınız	Hiçbir zaman (0 puan)	Bazen (1 puan)	Sıksık (2 puan)	Her zaman (3 puan)
*Duygusal Tepki*				
(1) Her ne kadar yardım edemesem de son dönemdeki krizi düşünmeden edemiyorum ve işin içinden çıkamıyorum.				
(2) Bana huzur veren şeylerin tadını tamamen kaybettim.				
(3) Mesafemi korumama rağmen insanlarla uğraşmaya tahammül edemiyorum, evde bile sinirliyim.				
(4) Yardımıma ihtiyacı olan birçok insanı ihmal ettiğimi hissediyorum.				
(5) Düşünmekte ve karar vermekte güçlük çekiyorum, birçok şüphem var, bir tür duygusal tıkanıklığa girdim.				
(10) Hastaların acı çekmesiyle empati kurmakta veya durumlarıyla bağlantı kurmakta güçlük çekiyorum (duygusal uzaklaşma, duygusal anestezi).				

*Korku ve Kaygı Cevabı*				
(6) İçinde olduğumuz kriz durumuyla tetiklenen yoğun fizyolojik reaksiyonlar (şok, terleme, baş dönmesi, nefes darlığı, uykusuzluk vb.) yaşıyorum.				
(7) Sürekli tetikte hissediyorum, tepkilerimin artık diğer hastaları, meslektaşlarımı veya kendimi riske attığına inanıyorum.				
(8) Hastalanmamak için endişelenmek bende dayanması zor bir gerginliğe neden oluyor.				
(9) Aileme hastalık bulaştıracağım diye korkuyorum.				

**Table 3:** Sociodemographic characteristics of the sample (*n* = 127).

	Mean ± SD (min–max)	Median (25th and 75th percentile)
Age	37.16 ± 8.42 (23–57)	37 (29–44)
Professional experience	13.97 ± 9.41 (1–38)	13 (5–21)
		*n* (%)
Age	20–30 years	37 (29.13%)
	30–40 years	38 (29.93%)
	40–50 years	41 (32.28%)
	>50 years	11 (8.66%)
Professional experience	0–5 years	35 (27.56%)
	5–10 years	19 (14.96%)
	10–20 years	40 (31.50%)
	>20 years	33 (25.98%)
Occupation	Doctor	34 (26.77%)
	Assistant doctor	28 (22.05%)
	Nurse/midwife/physiotherapist	60 (47.24%)
	Others (medical secretary, technical staff, security staff, etc.)	5 (3.94%)
Department of hospital	Emergency medical service and outpatient clinics	24 (18.90%)
	Intensive care units	15 (11.80%)
	Internal medicine units	46 (36.22%)
	Surgery units	21 (16.54%)
	Others (laboratory, information desk, etc.)	21 (16.54%)
COVID-19 disease history	Positive	32 (25.20%)
	Negative	95 (74.80%)
COVID-19 vaccination status	Positive	112 (88.19%)
	Negative	15 (11.81%)

SD: standard deviation; Min: minimum; Max: maximum; IQR: 25th and 75th percentiles.

**Table 4:** Intraclass correlation coefficients.

	ICC	95% confidence interval		ICC *p*	Intragroup *p*
		Lower bound	Upper bound		
Item 1	0.831	0.761	0.881	0.0001	0.110
Item 2	0.819	0.743	0.873	0.0001	0.071
Item 3	0.850	0.786	0.894	0.0001	0.753
Item 4	0.836	0.767	0.884	0.0001	0.239
Item 5	0.869	0.814	0.908	0.0001	0.739
Item 6	0.772	0.676	0.840	0.0001	0.743
Item 7	0.854	0.793	0.897	0.0001	0.832
Item 8	0.849	0.786	0.894	0.0001	0.148
Item 9	0.882	0.833	0.917	0.0001	0.058
Item 10	0.810	0.730	0.866	0.0001	0.842
Total	0.912	0.875	0.938	0.0001	0.116

ICC: intraclass correlation coefficient. The Wilcoxon signed rank test was used for intragroup examination.

**Table 5:** Item analysis of the EASE scale.

	Scale mean if item deleted	Corrected item-total correlation	Cronbach's alpha if item deleted
Item 1	8.93	0.600	0.880
Item 2	9.09	0.670	0.870
Item 3	8.99	0.700	0.870
Item 4	9.28	0.670	0.870
Item 5	9.38	0.740	0.870
Item 6	9.71	0.690	0.870
Item 7	9.73	0.660	0.870
Item 8	9.45	0.700	0.870
Item 9	8.65	0.390	0.890
Item 10	9.63	0.460	0.890

**Table 6:** Results of the confirmatory factor analysis based on the original version of the EASE scale.

Model fit indices	Score	Recommended cut-off value	
		Perfect fit	Acceptable fit
*Absolute fit indices*			
*χ* ^2^/df	1.826	0 ≤ *χ*^2^/df ≤ 2	2 ≤ *χ*^2^/df ≤ 3
GFI	0.905	0.95 ≤ GFI ≤ 1.00	0.90 ≤ GFI ≤ 0.95
AGFI	0.846	0.90 ≤ AGFI ≤ 1.00	0.85 ≤ AGFI ≤ 0.90
SRMR	0.0545	0 ≤ SRMR ≤ 0.05	0.05 ≤ SRMR ≤ 0.1

*Comparative fit indices*			
NFI	0.893	0.95 ≤ NFI ≤ 1.00	0.90 ≤ NFI ≤ 0.95
CFI	0.947	0.97 ≤ CFI ≤ 1.00	0.95 ≤ CFI ≤ 0.97

*Parsimonious fit indices*			
RMSEA	0.083	0 ≤ RMSEA ≤ 0.05	0.05 ≤ RMSEA ≤ 0.08
PGFI	0.56	0.95 ≤ PGFI ≤ 1.00	0.50 ≤ PGFI ≤ 0.95
PNFI	0.675	0.95 ≤ PNFI ≤ 1.00	0.50 ≤ PNFI ≤ 0.95
^∗^ *p* value = 0.002			

CFI: comparative fix index; GFI: goodness-of-fit index; AGFI: adjusted goodness-of-fit index; SRMR: standardized root mean square residual; NFI: normed fit index; CFI: comparative fit index; RMSEA: root mean square error of approximation; PGFI: parsimonious goodness-of-fit index; PNFI: parsimonious normed fit index. ^∗^*p* < 0.05 statistically significant model.

**Table 7:** Presentation of factor loads made with all items in the original EASE scale after the Turkish translation.

Stage 1		
Item	Factor (F1 = affective response; F2 = fear and anxiety response)	Estimate
Item 1	F1	0.651
Item 2	F1	0.721
Item 3	F1	0.761
Item 4	F1	0.718
Item 5	F1	0.806
Item 10	F1	0.502
Item 6	F2	0.786
Item 7	F2	0.736
Item 8	F2	0.777
Item 9	F2	0.397

**Table 8:** Evaluation of EASE subdimensions and score ranges in the Turkish validated scale.

	Score ranges and subdimensions	*n*	%
EASE score range	0–9: good emotional adjustment	60	47.24%
	10–14: emotional distress	40	31.50%
	15–24: emotional overload	25	19.69%
	≥25: extreme acute stress	2	1.57%
EASE subdimensions (mean ± SD; median (25th and 75th percentiles))	F1 = affective response	6.59 ± 3.54	6 (4–9)
	F2 = fear and anxiety response	3.72 ± 2.53	4 (1–5)
	Total score	10.31 ± 5.68	10 (6–14)

## Data Availability

The data supporting this research are available from the authors on reasonable request.
